# Willis and Sydenham: intersections and contrasts in early modern England

**DOI:** 10.1055/s-0046-1827164

**Published:** 2026-08-13

**Authors:** João Pedro Sá Lins, Rodrigo da Silva Sousa, Lucca Passow Carpinelli, Gustavo Leite Franklin, Hélio Afonso Ghizoni Teive, Alex Tiburtino Meira

**Affiliations:** 1 Programa de Residência em Neurologia, Escola de Saúde Pública da Paraíba, João Pessoa PB, Brazil.; 2 Hospital Metropolitano Dom José Maria Pires, Serviço de Neurologia, Santa Rita PB, Brazil.; 3 Institute of Public Health, University Hospital of Cologne, Cologne, Germany.; 4 Universidade Federal do Paraná, Setor de Ciências da Saúde, Departamento de Medicina Interna, Curitiba PR, Brazil.; 5 Pontifícia Universidade Católica do Paraná, Escola de Medicina e Ciências da Vida, Departamento de Medicina Interna, Curitiba PR, Brazil.; 6 Universidade Federal da Paraíba, Departamento de Medicina Interna, Serviço de Neurologia, João Pessoa PB, Brazil.; 7 Hospital Universitário Lauro Wanderley, Serviço de Neurologia, João Pessoa PB, Brazil.

**Keywords:** History of Medicine, History, 17th Century, Neurology, Neuroanatomy, Chorea

## Abstract

Thomas Willis and Thomas Sydenham exemplify contrasting modes of medical validation in Early Modern England shaped by political and institutional realignments after the English Civil War and the Stuart Restoration. Benefiting from his ties to the Crown and the Church of England, Willis combined anatomical investigation of the nervous system with iatrochemical and theological frameworks. In contrast, Sydenham, aligned with the Parliamentary cause and operating outside academic institutions, developed a clinical method grounded in systematic bedside observation and nosological description during London epidemics. Their trajectories reveal divergent medical epistemology that coalesced to influence the foundations of neurological reasoning and the modern clinical method.

## INTRODUCTION

The final decades of Early Modern England were marked by intense political and religious upheavals and transformations. The Restoration of the monarchy was marked by the repression of Puritan dissent and by the reconfiguration of political and academic networks of influence. 1 2 In this context, 2 physicians carried out activities of major intellectual impact: Thomas Willis (1621–1675), often regarded as the *founder of neurology* , 3 and Thomas Sydenham (1624–1689), known as the *English Hippocrates* . 4 5 While Willis represented the anatomical and iatrochemical approach associated with the academic circles of Oxford, Sydenham personified clinical empiricism in London. Their divergent trajectories reflect the tensions of the period. 6

## THOMAS WILLIS

**Figure 1:**
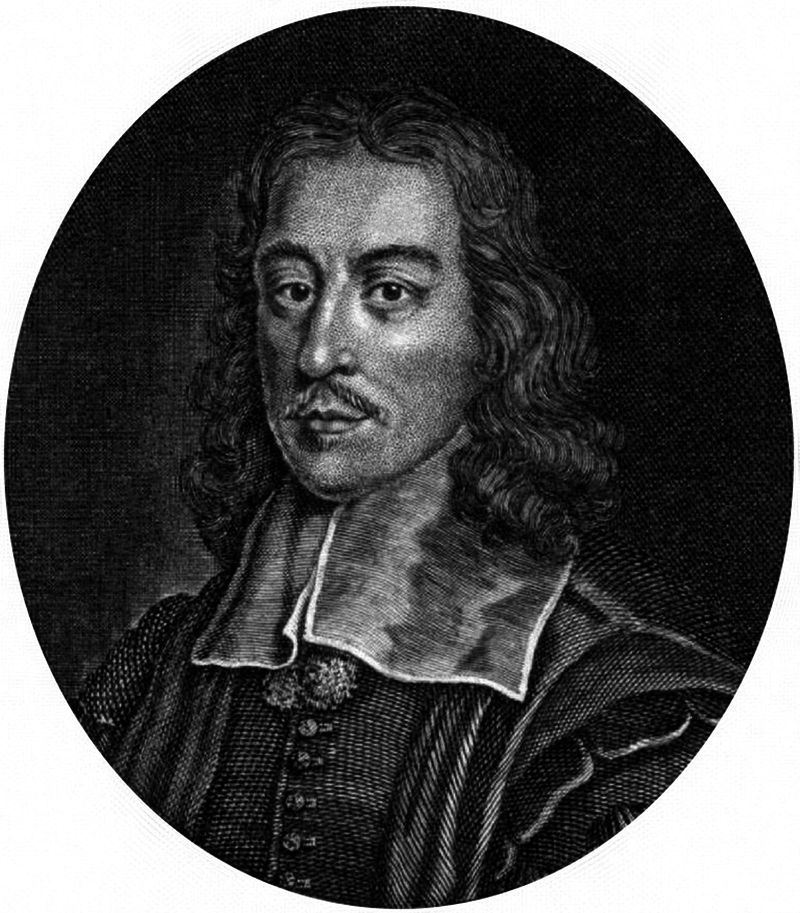
Thomas Willis (1621–1675). **Note:** Engraving by George Vertue, published in 1742, after an earlier portrait by David Loggan. Public domain.

Thomas Willis ( Figure 1 ) was born in Great Bedwyn, Wiltshire, in 1621, the son of a middling gentry family connected to local agriculture. He leaned towards an Anglican ecclesiastical career before his interests shifted to alchemy and medicine. 4 7 In the context of Civil War, he was appointed Bachelor of Medicine by royal decree after only 6 months of formal medical education. Initially, he struggled to establish himself professionally, but during the Restoration his political and religious alignment earned him important titles and social prominence, making him one of the most famous doctors of his time. 8 9

Originally oriented toward a religious vocation, Willis later embraced iatrochemistry before ultimately focusing on neuroanatomy. In *Cerebri Anatome* (1664), based on cadaver dissections, he described nervous structures, classified cranial nerves, mapped cerebral arteries, and demonstrated the anastomotic circle that bears his name. 7 10 This work reflected his Restorationist worldview, employing analogies that alluded to the Crown and the Church of England. 8 11 In dedication to the Archbishop of Canterbury, he stated that studying the brain would compel even ‘the most perverse atheist’ to ‘acknowledge God or refuse both religion and reason.’ 8 10

Thomas Willis is widely recognized as the first to coin the term *neurology* for the study of neurological diseases, and he made numerous discoveries in this field. Notably, in 1672, he described what is now recognized as *restless legs syndrome* . The disorder was later more precisely characterized by Karl-Axel Ekbom (1907–1977) and is currently referred to as Willis-Ekbom disease. 3 He also provided what is regarded as the earliest description of myasthenia gravis, reporting the case of a woman with fluctuating bulbar weakness with fatigability, an observation of remarkable historical significance. 3

In *Pathologiae Cerebri* (1667) and *De Anima Brutorum* (1672), Willis shifted toward an even more speculative epistemological model, describing *spiritus animalis* , volatile chemical substances that ascended through the cervical vessels and were transmitted via nerve fibers, explaining clinical conditions. 12

The reception of Willis's works ( Table 1 ) among his peers was ambivalent. *Cerebri Anatome* would become a landmark reference for European medicine in subsequent centuries. However, some of his philosophical extrapolations were dismissed by some colleagues as intellectual fantasy. 12 13

**Table 1:** Major works by Thomas Willis

Year	Original title	Commentary (article perspective)
**1659**	*Diatribae duae medico-philosophicae*	Focuses on fermentation and fevers. It reflects Willis's iatrochemical view, postulating that the organism is regulated by fermentation reactions.
**1664**	*Cerebri Anatome*	The author's magnum opus, detailing brain and nerve anatomy, notably described the ‘Circle of Willis’. The study featured a notable esthetic apparatus, including engravings by Christopher Wren. This neuroanatomical work is connected to a perspective rooted in theology and royalist ideology.
**1667**	*Pathologiae Cerebri et Nervosi Generis Specimen*	A study on brain pathology and nervous diseases like epilepsy. This work marks a shift toward a more speculative epistemological model.
**1672**	*De Anima Brutorum*	Explores the ‘soul of brutes’ and the mind-body relationship. It details *spiritus animalis* , volatile chemical substances transmitted via nerve fibers to explain clinical conditions.
**1674**	*Pharmaceutice Rationalis*	Systematizes pharmacology through a chemical and anatomical lens. It includes fundamental clinical observations, such as the sweet taste of urine in diabetic patients, enabling the later differentiation between diabetes mellitus and insipidus.
**1691**	*A Plain and Easy Method for Preserving... from the Plague*	A posthumous practical guide for combating the plague. It emphasizes pragmatic prevention, such as cleaning the air and strengthening the body against the ‘poison’ of the disease.

## THOMAS SYDENHAM

**Figure 2:**
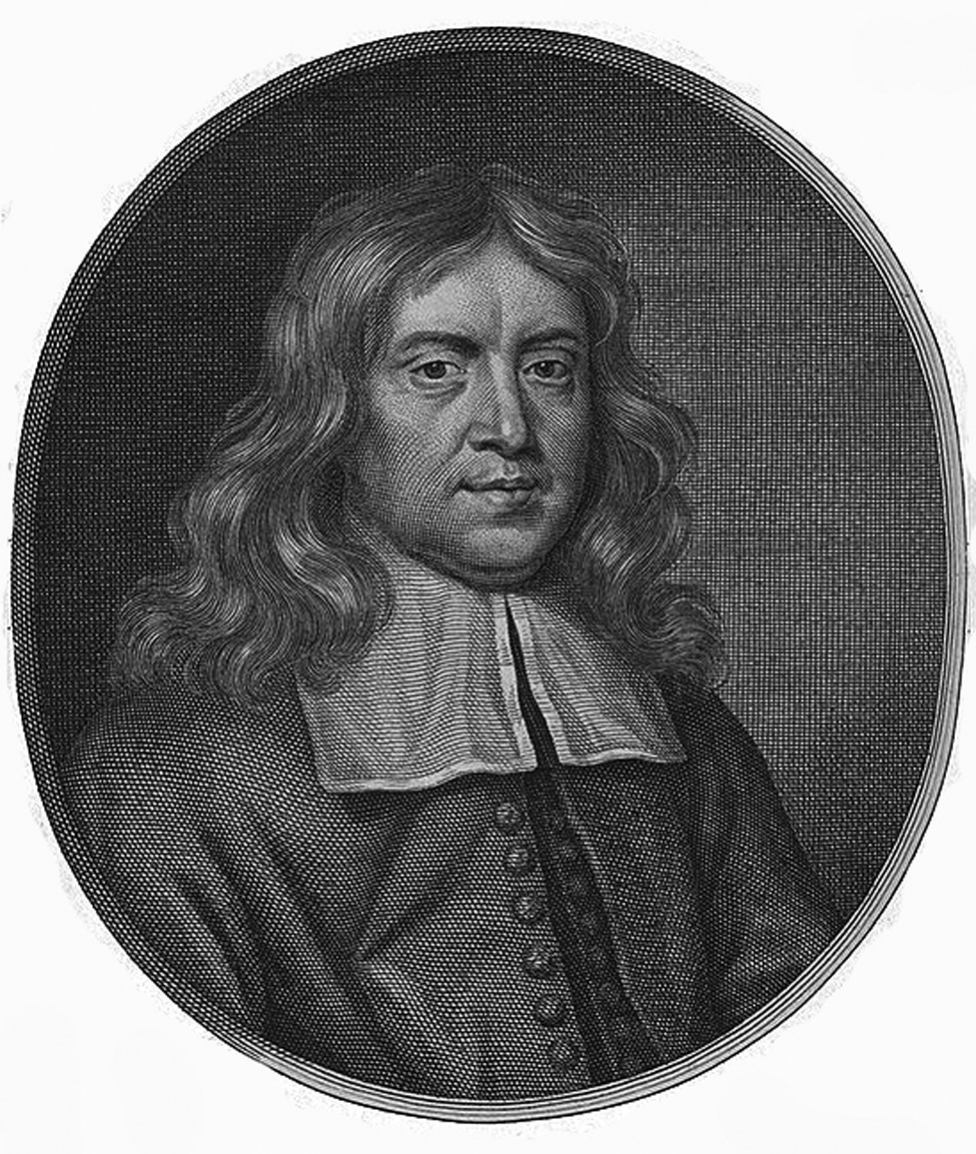
Thomas Sydenham (1624–1689). **Note:** Engraving by Abraham Blooteling, published in 1676, after an earlier portrait by Mary Beale. Public domain.

Thomas Sydenham ( Figure 2 ) was born in Wynford Eagle, Dorset, in 1624, into a Puritan family of the rural middling gentry with strong engagement to the Parliamentary side during the Civil War. In 1642, shortly after beginning his medical studies, he joined the Parliamentary Army. He later returned following the establishment of Puritan control and, in April 1648, obtained his medical degree by special warrant. 5 14 15 16

Under the influence of Baconian empiricism, he believed that the understanding of diseases should be derived from the close observation of signs and symptoms and the association of immediate causes, rather than from speculative theoretical models regarding hidden mechanisms. 15

His work was fundamental in taxonomically describing the natural history of diseases and in meticulously documenting health conditions and mortality rates in London during this period (1661–1675). Therefore, his writings are considered a foundational landmark in descriptive epidemiology. 17

In *Schedula Monitoria de Novae Febris Ingressa* , his late work, Sydenham provided the first precise clinical description of acute chorea, describing purposeless, irregular limb movements in children. His observation established chorea as a distinct clinical entity, later termed *Sydenham's chorea* . 18

In *Tractatus de Podagra et Hydrope* , Sydenham described gout in detail and related it to lifestyle factors, favoring dietary management over aggressive interventions such as bloodletting. The same empirical approach shaped his smallpox care, including a body-cooling method that drew criticism for defying Galenic, humoral-based treatments. 15 17

He was thus in conflict with the English medical establishment; however, his works' legacy ( Table 2 ) was secured by their dissemination throughout the European continent, primarily through his pupil and friend, John Locke. Later, 18th-century British physicians revisited his work and his contributions were recognized, earning him the title of *English Hippocrates* . 16

**Table 2:** Major works by Thomas Sydenham

Year	Original title	Commentary (article perspective)
**1666**	*Methodus Curandi Febres*	Sydenham's debut, introducing ‘bedside’ methodology, grounding his understanding of fevers in direct clinical observations rather than in philosophical theories.
**1676**	*Observationes Medicae*	His magnum opus. It expands his earlier work into a total system of ‘epidemic constitutions.’ He proposes that diseases are influenced by environmental and seasonal shifts, laying the foundations of nosology.
**1682**	*Dissertatio Epistolaris*	A letter-form treatise presenting Sydenham's clinical reasoning in an observational, practice-oriented register. It is also revolutionary for approaching hysteria as a widespread nervous condition rather than a strictly ‘uterine’ or mystical affliction.
**1683**	*Tractatus de* *Podagra et Hydrope*	A masterpiece of clinical description regarding gout and dropsy. Based on his own 30-year struggle with the disease, Sydenham provides a narrative so precise it remains a classic of medical literature.
**1686**	*Schedula Monitoria de Novae Febris Ingressu*	A late ‘practical memorandum’ on the approach to a newly-emergent fever, notable for containing Sydenham's classic clinical description of chorea minor (St. Vitus' dance/Sydenham's chorea).

## THE DISPARITIES

The 17th-century English crisis of authority also challenged established Hippocratic and Galenic medical foundations. 19 Within this legitimacy vacuum, Willis and Sydenham sought new forms of validations for medical knowledge, establishing divergent epistemological traditions that reflected their respective political and confessional alignments. 17 Willis, an Anglican Royalist, projected a hierarchical and theological order onto his neuroanatomy. Conversely, Sydenham's empiricism, centered on therapeutic efficacy, resonated with the Puritan ethos.

This distinction also manifested stylistically. Willis's *Cerebri Anatome* , with its engravings and dedications, used an esthetic apparatus and patronage networks to monumentalize his theory. 10 In contrast, Sydenham's direct and sober prose reflected his clinical method. 15 17 19

Evidence suggests that their divergences were already pronounced at the time, as attested by contemporary written records. ‘Sydenham and some others in London say of Dr. Willis that hee is an ingenious man but not a good physitian, and that hee does not understand the way of practice.’ 14 Willis plausibly ignored these attacks to avoid granting visibility to an outsider's critiques.

Nevertheless, rather than mutually-exclusive failures, these disparities were productive tensions. Willis's anatomical and terminological innovations supplied neurology's structural knowledge, while Sydenham's nosological framework and methodological rigor shaped clinical reasoning and epidemiology. Their opposition ultimately helped found modern neurology and neuroscience.

In conclusion, comparing these figures reveals the non-linear trajectory of medical epistemology, highlighting the interplay between Willis's experimental approach and Sydenham's clinical empiricism, and it demonstrates how these divergent traditions established the foundations of the modern clinical method and influenced seminal British neurologists in subsequent generations.

## References

[1] Hill C The Century of Revolution, 1603–1714 Abingdon, UK Routledge 1980

[2] Shapin S A Social History of Truth: Civility and Science in Seventeenth-Century England Chicago University of Chicago Press 1994 11620329

[3] Teive H AG Coutinho L Camargo C HF Munhoz R P Walusinski O Thomas Willis' legacy on the 400th anniversary of his birth Arq Neuropsiquiatr 2022 80 07 759 762 10.1055/s-0042-1755278 36254448 PMC9685818

[4] Feindel W Thomas Willis (1621-1675)-The Founder of Neurology Can Med Assoc J 1962 87 06 289 296 20327202 PMC1849485

[5] Dewhurst K THOMAS SYDENHAM (1624-1689) REFORMER OF CLINICAL MEDICINE Med Hist 1962 6 02 101 118 10.1017/S0025727300027083 16562238 PMC1034697

[6] Wear A Knowledge and Practice in English Medicine, 1550–1680 Cambridge Cambridge University Press 2000

[7] Arráez-Aybar L A Navia-Álvarez P Fuentes-Redondo T Bueno-López J L Thomas Willis, a pioneer in translational research in anatomy (on the 350th anniversary of Cerebri anatome) J Anat 2015 226 03 289 300 10.1111/joa.12273 25688933 PMC4337668

[8] Caron L Thomas Willis, the Restoration and the First Works of Neurology Med Hist 2015 59 04 525 553 10.1017/S0025727315001050 26352303 PMC4595957

[9] Clericuzio A Thomas Willis' iatrochemistry and the activity of matter Notes Rec R Soc Lond 2023 77 04 712 732 10.1098/rsnr.2023.0025

[10] O'Connor J Thomas Willis and the background to Cerebri Anatome Proc R Soc Med 2003 96 03 139 143 10.1258/jrsm.96.3.139PubMed PMC539424 12612118

[11] Harley D N Medical metaphors in English moral theology, 1560-1660 J Hist Med Allied Sci 1993 48 04 396 435 10.1093/jhmas/48.4.396 7506719

[12] Eadie M J A pathology of the animal spirits – the clinical neurology of Thomas Willis (1621-1675) part I – background, and disorders of intrinsically normal animal spirits J Clin Neurosci 2003 10 01 14 29 10.1016/S0967-5868(02)00165-0 12464516

[13] Wragge-Morley A. Imagining the soul: Thomas Willis (1621–1675) on the anatomy of the brain and nerves. Prog Brain Res 2018;243:55–73 10.1016/bs.pbr.2018.10.009PubMed 30514530

[14] Dewhurst K Dr. Thomas Sydenham (1624-1689): His Life and Original Writings London Wellcome Historical Medical Library 1966

[15] Bates D G Sydenham and the medical meaning of “method” Bull Hist Med 1977 51 03 324 338 334288

[16] Anstey P The creation of the English Hippocrates Med Hist 2011 55 04 457 478 10.1017/s0025727300004944 22025796 PMC3199640

[17] French R Wear A, editors.The Medical Revolution of the Seventeenth Century Cambridge Cambridge University Press 1989

[18] Vale T C Cardoso F Chorea: A Journey through History Tremor Other Hyperkinet Mov (N Y) 2015 5 tre-5-296 10.7916/D8WM1C98PubMed PMC4454991 26056609

[19] Cook H J The Decline of the Old Medical Regime in Stuart London Ithaca, NY Cornell University Press 1986

